# Field efficacy of a new deltamethrin long lasting insecticidal net (LifeNet©) against wild pyrethroid-resistant *Anopheles gambiae* in Benin

**DOI:** 10.1186/s12889-018-5876-9

**Published:** 2018-08-02

**Authors:** Armel Djènontin, Nicolas Moiroux, Aziz Bouraïma, Barnabas Zogo, Ibrahim Sidick, Vincent Corbel, Cédric Pennetier

**Affiliations:** 1 Faculté des Sciences et Techniques-Université d’Abomey-Calavi, Cotonou, Benin; 2 MIVEGEC, IRD, CNRS, Univ Montpellier, Cotonou, Benin; 3 Centre de Recherche Entomologique de Cotonou (CREC), Cotonou, Benin; 4 MIVEGEC, IRD, CNRS, Univ Montpellier, Montpellier, France; 5 IRSS, CNRST, Bobo Dioulasso, Ouagadougou, Burkina Faso; 6 IPR, INSP, Bouaké, Côte d’Ivoire

**Keywords:** Deltamethrin, Long lasting insecticidal net, LifeNet©, Pyrethroid-resistant, *Anopheles gambiae*, Benin

## Abstract

**Background:**

Malaria vector control is mostly based on Long-Lasting Insecticidal Nets (LLIN). To date, all LLINs fully recommended by the World Health Organization Pesticide Scheme (WHOPES) are made of polyester or polyethylene. In this context, a new LLIN named LifeNet©, made of polypropylene fiber is developed. According to the manufacturer, LifeNet©is made of soft filament, has a greater mechanical strength, a superior insecticide wash resistance with a short insecticide regeneration time, a better flammability profile and a better environmental profile compared to polyester or polyethylene nets.

**Methods:**

Through a WHOPES supervised trial, the efficacy of LifeNet© was evaluated in Benin in experimental huts against free-flying wild mosquitoes.

**Results:**

LifeNet© has equal or better performances in terms of wash resistance, exophily, blood feeding inhibition and mortality compared to conventionally treated nets (CTN) treated with deltamethrin at 25 mg/m^2^ and washed to just before exhaustion.

**Conclusions:**

The efficacy of LifeNet© observed in this trial indicates that this net fulfill World Health Organization Pesticide Scheme (WHOPES) requirement for Long Lasting technology in Phase II. Throughout a Phase III trial currently ongoing in Southern Benin, the durability and the acceptability of this long-lasting insecticidal mosquito nets will be assessed under community conditions.

## Background

Malaria is an entirely preventable and treatable disease. Its represents one of the most critical public-health challenges for Africa [1, 2]. In the absence of an effective vaccine, the World Health Organization (WHO) recommends prompt access to diagnosis, artemisinin-based combination therapy, long-lasting insecticidal nets (LLIN), indoor residual spraying of insecticide (IRS) and intermittent preventive treatment during pregnancy. Since 2000, there has been a tremendous increase in the financial support for malaria control. Therefore, malaria control programs have implemented heavily malaria vectors control tools such as the massive distribution of long-lasting insecticidal nets (LLIN) and indoor residual spraying of insecticide (IRS). The percentage of households owning at least one long-lasting insecticidal nets (LLIN) in sub-Saharan Africa increased from 3% in 2000 to 79% in 2015 making it the most widely deployed vector control tool in sub-Saharan Africa [1, 2]. Regarding IRS, the percentage of people protected by this intervention in the African Region increased from less than 5% in 2005 to 11% in 2010 but declined since 2011. Scaling up of malaria vector control has led to a considerable reduction in malaria incidence and mortality [1, 2]. However, despite this major decrease of the malaria burden, the disease is still of major public health concern, with an estimated 212 million cases and 429,000 deaths in 2015 [2]. Household surveys indicate that 96% of persons with access to an LLIN use it [1]. Nevertheless, this number might overestimate the real LLINs use [3]. For example in Benin, the real use of LLINs was showed to be less than 50%, even in the framework of a controlled trial [4]. Among reasons of low LLIN use is the discomfort of having to sleep under an LLIN when nocturnal temperature are high [5]. Net fabric may play a role in the comfort when using it as well as wash resistance and these factors could influence net use rates. To date, all LLINs fully recommended by the World Health Organization Pesticide Scheme (WHOPES) are made of polyester or polyethylene [6]. Polyester nets are usually smooth and soft to the touch with good user acceptance, but generally lack high mechanical strength, rarely maintaining their physical integrity beyond 2–3 years. Polyethylene nets are generally more resistant than polyester ones. However, they often need heat treatments or extra time for insecticide regeneration and are usually rough to the touch, contributing to lower acceptance [6].

Pyrethroid LLINs are the main LLINs recommended by the WHOPES because of their strong efficacy, their fast acting effect at low dose, and their low toxicity for mammals [7]. In countries where LLINs were implemented at large scale, malaria vectors have developed resistance to pyrethroids [8]. However until now, there is no evidence that pyrethroid resistance reduced the effectiveness of LLINs for controlling malaria at an operational level. Moreover, since the advent of pyrethroids in the 1970s, very few or no new major class of active ingredients (AI) has appeared in the pipeline of public-health products. Suppliers estimate that developing a new AI today takes at least 10 years and its cost might reach $300 million [9]. Then, the development of new LLINs is therefore based on existing pyrethroids used alone or in combination with synergist or Insect Growth Regulator to impregnate polyethylene, polyester or alternative materials [10, 11].

In this context, Bayer Environmental Science developed a new LLIN named LifeNet©. The LifeNet® is a deltamethrin-treated LLIN. Technical deltamethrin is incorporated into 100 denier poly-filament polypropylene fibers at the target dose of 8.5 g AI/kg, corresponding to 340 mg of deltamethrin per m^2^. According to the manufacturer, this new LLIN is made of soft filament, has a greater mechanical strength, a superior insecticide wash resistance with a short insecticide regeneration time, a better flammability profile and a better environmental profile compared to polyester or polyethylene nets. Here, we evaluated the efficacy of this new Long Lasting Insecticidal Net in experimental huts against free-flying wild mosquitoes under WHOPES supervision.

## Methods

### Study area

The study area is located in Malanville, a District of northern Benin, situated in a Sudanian savannah area, near rice fields. This field station belongs to the *Anopheles* Biology and Control (ABC) Network [10]. The area is characterized by a long dry season lasting from December to June. An irrigation system from the Niger River allows rice cultivation during the dry season. *Anopheles gambiae s*.*l*. is the main malaria vector with 95.7% *Anopheles coluzzii*, and 3.5% *Anopheles arabiensis* [12, 13]*.* In this area, *An. gambiae s.l* was showed previously to be susceptible to most pyrethroids [12, 14]. However, a significant increase of pyrethroid resistance (< 50% mortality at 0.05% deltamethrin) due to the occurrence of the *kdr* mutation (frequency of 1014F allele = 47%) and enhanced oxidase activity was observed at the time of the present evaluation [15].

### Design of the huts

**Fig. 1:**
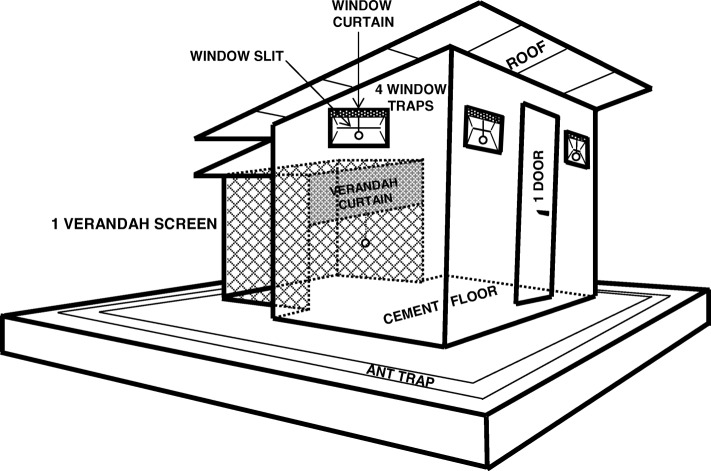
Design of the experimental huts commonly used in West Africa

Experimental stations are composed of several identical experimental huts designed to resemble local housing. The walls are made with concrete bricks, the ceiling with a polyethylene sheeting and the roof with iron. The huts are surrounded by water-filled channel in order to avoid the entry of ants. (Fig. 1). Each experimental hut have four windows made with metal pieces which are placed with an angle creating a 1 cm gap so as to allow mosquitoes to entry easily and to limit their exit from the hut. At the back of each experimental hut a veranda trap 1.5 m high, 1.5 m wide and 2 m long, is built. This veranda is made with a polyethylene sheeting. During the night, mosquitoes can move, as well as, from the hut to the veranda and from the veranda to the hut (Fig. 1) [16].

### Treatments arms

Washed and unwashed LifeNet© LLINs were evaluated during the study. The negative control was an untreated net. The insecticide treated nets used as reference were two polyester nets conventionally impregnated with deltamethrin at 25 mg/m^2^ (CTN) washed to just before exhaustion and washed 20 times. The nets were conventionally treated at CREC in Cotonou, Benin. The CTN were washed according to the WHOPES procedures in order to determine the point of exhaustion [17]. Washing were carried out on a 1 day interval [18]. After each wash, WHO standard cone test were done. The number of washes after which the CTN still induced 80% mortality or 95% KD was the number of washed required before exhaustion.

The treatment arms tested were the follow:

1. Unwashed deltamethrin long lasting insecticidal net named LifeNet©;
2. Deltamethrin long lasting insecticidal net named LifeNet© washed 20 times;
3. Deltamethrin long lasting insecticidal net named LifeNet© washed 30 times;
4. Polyester net conventionally impregnated with deltamethrin at the dose 25 mg Al/m^2^ (CTN) washed to just before exhaustion;
5. 20 times washed polyester net conventionally impregnated with deltamethrin at the dose 25 mg Al/m^2^ (CTN);
6. Polyester net with same mesh size as LifeNet© and without any insecticide.

This study had lasted for 12 weeks corresponding to two complete Latin squares. Each week, a rotation of the treatment arms was done among the huts according to a Latin square scheme. Per treatment, six nets were used. Each of the six nets was used only one night during a week a (the evaluation was run 6 days per week). All huts were carefully cleaned and ventilated at the end of each week in order to remove eventual pesticides residues. Considering the six treatment arms, 6 weeks were needed to ensure a complete rotation of treatment arms and sleepers among huts. To obtain sufficient numbers of mosquitoes for statistical analysis, two Latin square were needed. According to the WHOPES procedures, all nets to be tested were deliberately holed (six holes on each net, two holes in each of the long sides and one hole in each of the others sides. Each hole measures 4 cm × 4 cm [17].

### Volunteer participants and mosquito collections

The participants to the study were adult volunteers recruited among the inhabitants of the villages close to the experimental station. The selection was done after having received the approval of the local authorities. The volunteer participants (sleepers) were informed on the objectives of the study and they have signed an informed consent (written in English and French). Before the beginning of the experimental hut trial, each sleeper received a medical certificate by the physician of the Malanville health centre to authorize the work. The sleepers entered the huts at 8:00 PM and remained inside until the morning at 6:00 AM. In the morning, volunteer participants collected mosquitoes in the huts and on the veranda using mouth aspirators. Females mosquitoes collected alive, dead, fed or unfed in the hut and on the veranda were counted and kept separately. Alive female mosquitoes were fed with sugar solution for 24 h for assessing mortality after 24 h (delayed mortality).

The entomologic indicators measured in the experimental huts were:

- the reduction in female mosquitoes hut entry relative to the control (deterrence);
- the proportion of female mosquitoes collected on the veranda (exophily);
- the reduction in female mosquitoes blood feeding relative to the control hut (blood feeding inhibition);
- the proportion of female mosquitoes found dead in the morning and after 24 h (immediate mortality and delayed mortality);

The personal protection (*pp*) effect of a treated net and their killing effect (*ke*) were also estimated as follows:

$$ pp\left(\%\right)=100\times \left( bu- bt\right)/ bu $$

where *bu* is the total number of blood-fed female mosquitoes in the negative control hut and *bt* is the total number of blood-fed female mosquitoes in the huts with insecticide treated nets.

$$ ke\left(\%\right)=100\times \left( kt- ku\right)/ tu $$

where *kt* is the total number of female mosquitoes killed in the huts with insecticide treated nets, *ku* is the total number of female mosquitoes killed in the negative control hut and *tu* is the total number of female mosquitoes collected from the negative control hut.

### Bioassays

Six randomly selected nets to be used in the huts (one per treatment arm: 1 untreated net, 3 LifeNets and 2 CTNs) were bio-assayed before any washing, after washings and after field experiments. Standard WHO cone tests were done [19]. Five cones were placed on each net on the 5 sides. Under each cone, 5 females of *An. gambiae* susceptible reference strain were introduced for 3 min. Bioassays were replicated two times per side (10 females mosquitoes per cone) to ensure that 50 mosquitoes in overall were tested per net. Knock Down (KD) was checked after 60 min and the mortality 24 h after exposition was recorded. The cone test was done after each wash for the CTN washed to just before exhaustion and until mortality and KD decreased under 80% et 95% respectively. Then washes were stopped.

### Side effects perceived by the sleepers

After sleeping in each treatment arm, the sleepers were questioned in order to record beneficial and adverse effects they perceived during the evaluation in each treatment.

### Data analysis

The non parametric Kruskal-Wallis test was used for analysis of entry rates. Proportional data (exophily, blood feeding, mortality) were analyzed using logistic regression. Each treatment was successively used as the reference class for multiple comparisons. One analysis has been run on the *An. gambiae s.l.* collected in the huts and another analysis has been run on all mosquitoes collected. Data of mortality and KD recorded with the bioassays were compared between treatment arms using a χ^2^ test. Analysis were done using STATA 6 Software (Stata Corporation, College Station, TX, USA).

## Result

### Bioassays

#### Point of exhaustion and initial bioefficacy of the treated nets

Before any washing, all treated nets were effective in terms of KD effect and mortality (Table 1). After 3 washes of the CTN, KD and mortality decreased below the WHO threshold (95% KD or 80% mortality). KD and mortality were 85 and 77% respectively. Hence 2 washes were considered as the maximum number of washes required to be just before exhaustion (Table 2).

**Table 1:** Knockdown and mortality of susceptible *An. gambiae* (Kisumu strain) recorded after 3 min exposure under WHO cones on nets before any washing

Treatments	N mosquitoes tested	% KD after 60 min	% Mortality after 24 h
Untreated polyester net	52	0^a^	0^a^
Life Net to be unwashed	54	100^b^	100^b^
Life Net to be washed 20 times	53	100^b^	100^b^
Life Net to be washed 30 times	54	100^b^	100^b^
CTN 25 mg/m^2^ to be washed just before exhaustion	53	100^b^	100^b^
CTN 25 mg/m^2^ to be washed 20 times	57	100^b^	100^b^

*CTN* conventionally deltamethrin-treated nets. Values in the same column sharing different letter superscript differ significantly (χ^2^ = 101, df = 1, *P* = 0.000)

**Table 2:** Knockdown and mortality of susceptible *An. gambiae* (Kisumu strain) recorded after 3 min exposure under WHO cones on nets conventionally treated with deltamethrin at 25 mg/m^2^ (CTN) and submitted to successive washes

Number of washes	N mosquitoes tested	% KD after 60 min	% Mortality after 24 h	Control % Mortality after 24 h (n)
0 wash	53	100	100	0 (51)
1 wash	52	98	90	0 (54)
2 washes	54	93	83	0 (53)
3 washes	52	**85**	**77**	0 (56)

After all washes were completed and before being installed in the experimental huts, LifeNet© unwashed, washed 20 times and washed 30 times gave similar results in term of KD effect (100%) and mortality (100%). A significant decrease of mortality (χ^2^ = 4.00 and 6.36, *N* = 53 and 50, df = 1, *p* = 0.045 and 0.012) was observed for the CTN washed just before exhaustion and the CTN washed 20 times (92 and 74% mortality respectively) (Table 3).

**Table 3:** Knockdown and mortality of susceptible *An. gambiae* (Kisumu strain) recorded after 3 min exposure under WHO cones on nets after washing and before field testing

Treatments	N mosquitoes tested	% KD after 60 min	% Mortality after 24 h
Untreated polyester net	51	0^a^	0^a^
Life Net unwashed	53	100^b^	100^b^
Life Net washed 20 times	50	100^b^	100^b^
Life Net washed 30 times	51	100^b^	100^b^
CTN 25 mg/m^2^ washed just before exhaustion	53	94^b^	92^c^
CTN 25 mg/m^2^ washed 20 times	50	94^b^	74^d^

*CTN* conventionally deltamethrin-treated nets. Values in the same column sharing different letter superscript differ significantly (χ^2^ = 92.66, 4.00 and 6.36, df = 1, *P* = 0.000, 0.045 and 0.012)

#### Bioefficacy of the treated nets after field testing

At the end of the field trial, no significant difference of KD effect was noted between LifeNet© (unwashed and regardless of the washing regimen) and CTN washed just before exhaustion. KD effect of CTN washed 20 times was significantly lower (χ^2^ = 6.04, *N* = 57, df = 1, *p* = 0.014). Mortality induced by LifeNet©, regardless of the washing regimen, was significantly higher (χ^2^ = 12.42, *N* = 53, df = 1, *p* = 0.000) than the mortalities induced by the CTNs (Table 4).

**Table 4:** Knockdown and mortality of susceptible *An. gambiae* (Kisumu strain) recorded after 3 min exposure under WHO cones on nets after washing and after field testing

Treatments	N mosquitoes tested	% KD after 60 min	% Mortality after 24 h
Untreated polyester net	49	0^a^	0^a^
Life Net unwashed	52	100^b^	100^b^
Life Net washed 20 times	54	100^b^	100^b^
Life Net washed 30 times	60	100^b^	100^b^
CTN 25 mg/m^2^ washed just before exhaustion	53	94^b^	81^c^
CTN 25 mg/m^2^ washed 20 times	57	63^c^	60^d^

*CTN* conventionally deltamethrin-treated nets. Values in the same column sharing different letter superscript differ significantly (χ^2^ = 101, 15.65 and 6.04, df = 1, *P* = 0.000 and 0.014)

#### Efficacy under experimental huts

The evaluation was run between the 28th November 2010 and the 1st April 2011. Nets were evaluated during 72 collection nights per hut, i.e. 6 nights per week during 12 weeks (two complete Latin squares). We interrupted collections during 6 weeks (from 10th January to 20th February 2011) due to the lack of mosquitoes during the peak of the dry season. In total, 445 females *An. gambiae s.l.* and 4481 females of other mosquitoes were collected during the trial (Tables 5 and 6).

**Table 5:** Summary results obtained for free flying wild culicidae (72 nights) in experimental huts

	Untreated Net	LifeNet 0 wash	LifeNet 20 washes	LifeNet 30 washes	CTN 25 mg/m^2^ Exhaust	CTN 25 mg/m^2^ 20 washes
Total females caught	689^ab^	871^a^	704^ab^	654^b^	825^ab^	738^ab^
females caught/night	9.-56	12.-09	9.-77	9.-08	11.-45	10.-25
Deterrency (%)	–	−26	−2	5	−20	−7
Total females veranda	231	338	309	260	276	339
Exophily (%)	34^a^	39^b^	44^bc^	40^b^	33^a^	46^c^
95% Confidence limits	30–37	36–42	40–48	36–44	30–37	42–50
Induced Exophily (%)	–	16	31	19	NS	37
Total females blood fed	224	12	13	15	40	79
Blood fed (%)	33^a^	1^c^	2^c^	2^c^	5^b^	11^d^
95% Confidence limits	29–36	1.-2	1.-3	1.-3	3.-6	8.-13
Blood feeding inhib. (%)	–	96	94	93	85	67
Total females dead	79	846	662	599	687	440
Overall mortality (%)	11^a^	97^c^	94^d^	92^d^	83^b^	60^e^
95% Confidence limits	9.-14	96–98	92–96	89–94	81–86	56–63
Corrected for control (%)	–	97	93	91	81	54

*CTN* conventionally deltamethrin-treated nets. Values in the same column sharing different letter superscript differ significantly (logistic regression, *P* = 0.00)

**Table 6:** Summary results obtained for free flying wild *Anopheles gambiae* (72 nights) in experimental huts

	Untreated Net	LifeNet 0 wash	LifeNet 20 washes	LifeNet 30 washes	CTN 25 mg/m^2^ Exhaust	CTN 25 mg/m^2^ 20 washes
Total females caught	87^a^	65^a^	69^a^	76^a^	62^a^	86^a^
females caught/night	1.20	0.90	0.96	1.05	0.86	1.19
Deterrency (%)	–	25	21	13	29	1
Total females veranda	13	36	41	47	33	33
Exophily (%)	15 ^a^	55 ^b^	59 ^b^	62 ^b^	53 ^bc^	38 ^c^
95% Confidence limits	7–22	43–67	48–71	51–73	41–66	28–49
Induced Exophily (%)	–	271	298	314	256	157
Total females blood fed	33	3	1	8	9	26
Blood fed (%)	38 ^a^	5 ^bc^	1^c^	11^b^	15^b^	30^a^
95% Confidence limits	28–48	0–10	0–4	4.-17	6.-23	21–40
Blood feeding inhib. (%)	–	88	96	72	62	20
Total females dead	1	49	48	42	27	22
Overall mortality (%)	1^a^	75^c^	70^cd^	55^bd^	44^b^	26^e^
95% Confidence limits	0–3	65–86	59–80	44–66	31–56	16–35
Corrected for control (%)	–	75	69	55	43	25

*CTN* conventionally deltamethrin-treated nets. Values in the same column sharing different letter superscript differ significantly (logistic regression, *P* = 0.00)

#### Deterrence

During the 72 nights of collection, 87 females *An. gambiae s.l.* and 689 females of other mosquitoes were collected in the control hut. The mean numbers of females mosquitoes caught per night in the control hut were 9.6 for other mosquitoes and 1.2 for females *An. gambiae s.l..*. In the treated huts, the number of females other mosquitoes collected ranged from 654 in the hut with the LifeNet© washed 30 times to 871 in the hut with the unwashed LifeNet© (Table 5). Regarding females *An. gambiae s.l.*, the number caught ranged from 62 in the hut with the CTN washed to just before exhaustion to 86 in the hut with the CTN washed 20 times (Table 6). There was no significant difference in entry rates either for other mosquitoes or *An. gambiae s.l.* between the treated nets and the control.

#### Exophily

Exophily in the control hut was 34% for other mosquitoes and 15% for *An. gambiae s.l*. In the treated huts, exophily of other mosquitoes ranged from 33% (in the hut with the CTN washed to just before exhaustion) to 46% (in the hut with the CTN washed 20 times). Regarding *An. gambiae* s.l., exophily ranged from 38% (in the hut with the CTN washed 20 times) to 62% (in the hut with the LifeNet© washed 30 times) (Tables 5 and 6).

#### Blood feeding

Twenty-three percent of other mosquitoes and 38% of the *An. gambiae s.l*. collected during the trial in the control hut were blood fed. This corresponds to an average of 3.2 other mosquitoes bites and 0.46 *An. gambiae s.l*. bites per person per night. Except for the CTN washed 20 times, all treatments significantly inhibited blood feeding (logistic regression, *p* = 0.000) (other mosquitoes and *An. gambiae s.l*.) compared to the control (Tables 5 and 6). It is interesting to note that the LifeNet© washed 20 times and 30 times induced greater blood feeding inhibition (96 and 72%) against malaria vectors than the CTN washed just before exhaustion (62%) and the CTN washed 20 times (20%) (Table 6). The same trend was observed with the other mosquitoes (Table 5).

The personal protection against other mosquitoes bites ranged from 95% with unwashed LifeNet© to 65% with a CTN washed 20 times (Table 5). Regarding personal protection against *An. gambiae s.l*. bites, it ranged from 97% with LifeNet© washed 20 times to 21% with CTN washed 20 times (Table 6). After 30 washes, personal protection conferred by LifeNet© against *An. gambiae s.l*. bites (76%) was nearly 4 times higher than that conferred by CTN washed 20 times (21%) (Table 6).

#### Mortality

Mortality rate of females of other mosquitoes in the control hut was 11% and that of females *An. gambiae* s.l. was 1%. All treatments caused significantly higher mortality than the control arm (*P* < 0.001). The LifeNet© unwashed and washed 20 times induced equal or greater mortality than the CTN washed to just before exhaustion (*P* < 0.05). Interestingly, the mortality rates induced on females of other mosquitoes and females *An. gambiae s.l.* by LifeNet© washed 20 times and 30 times were similar. The CTN washed 20 times killed significantly lower number of other mosquitoes and *An. gambiae* s.l. compared to all other treatments (logistic regression, *p* = 0.000).

The killing effect against other mosquitoes ranged from 100% with unwashed LifeNet© to 52% with CTN washed 20 times (Table 5). Regarding the killing effect against *An. gambiae s.l*., it ranged from 55% with unwashed LifeNet© to 24% with CTN washed 20 times (Table 6). After 30 washes, the killing effect induced by LifeNet© against *An. gambiae s.l*. (47%) was nearly 2 times higher than that induced by CTN washed 20 times (24%)(Table 6).

#### Side effects

No perceived adverse effects were noted by the 6 volunteers regardless of the treatment.

## Discussion

In experimental huts, LifeNet© showed good performances in terms of exophily, blood feeding inhibition and mortality, against wild *An. gambiae s.l*. and against other mosquito population.

Before any washing, all treated nets induced 100% mortality. Such results show that deltamethrin was bio-available for mosquitoes. Bio-efficacy of LifeNet© was high compared to polyester CTN. The wash resistance of LifeNet©, as measured using bioassays, was equal or greater than that of other LLINs recommended by WHOPES [11, 20–24]. The wash resistance of LifeNet© could be explained by the higher dosage of the insecticide incorporated into the polypropylene fibers or by the incorporation technology. It is also possible that interactions between polypropylene fibers and insecticide explains this high wash resistance. Indeed, high wash resistance of insecticide-treated polypropylene has been observed previously, possibly due to an interaction between this material and the insecticide [25]. The wash resistance of LifeNet© is promising for the long term use of this net at community level and it should be tested under such conditions.

*An. gambiae s.l*. mortality obtained in the control arm (untreated net) was low (1%) and similar to that previously observed with untreated holed mosquito nets [10, 11, 26]. This is the indication that the study design is reliable and no contamination has occurred during the rotation of the treatments among huts. Exophily of *An. gambiae* recorded during this study in the control arm was lower than that observed in previous experimental hut trials conducted in Malanville (15% here versus 35 to 45% in [10]). All treated nets induced significantly higher exophily rates than the control (from 16 to 37%) for the whole mosquito population collected in the huts. Surprisingly for *An. gambiae*, a very high induced exophily was observed regardless of the treatment arms (157 to 314%). This could be explained by the low exophily rate recorded in the control hut for this species. Previous studies have shown that female mosquitoes may look for suitable resting sites in order to use the nutritive value of the blood meal to survive until the end of the dry season [27]. Since the trial has been run in the middle of the dry season, the induced exophily trend observed may be explained by this behaviour of *An. gambiae* in dry season and highlights the importance to consider the season when conducting experimental hut evaluation of insecticide treated materials.

Despite sudden increase of pyrethroid-resistance in malaria vectors in Malanville [15], this study showed that the performance (i.e. blood feeding inhibition and mortality) of LifeNets washed 20 and 30 times was equal or higher than that of a CTN washed to just before exhaustion. The results of this trial showed that the resistance does not seem to be a major obstacle for the evaluation of LLIN products.

## Conclusions

The efficacy of LifeNet© observed during this trial indicates that this net fulfill World Health Organization Pesticide Scheme (WHOPES) requirement for Long Lasting technology in Phase II. Throughout a Phase III trial currently ongoing in southern Benin, the durability and the acceptability of this long-lasting insecticidal mosquito nets will be assessed under community condition.

## Abbreviations

ABC: *Anopheles* Biology and Control Network
AI: Active Ingredients
CTN: Conventionally Treated Nets
IRS: Indoor Residual Spraying
*kdr*: Knock Down Resistance
LLIN: Long-Lasting Insecticidal Nets
WHO: World Health Organization
WHOPES: World Health Organization Pesticide Scheme
